# Slower clozapine titration is associated with delayed onset of clozapine-induced fever among Japanese patients with schizophrenia

**DOI:** 10.1038/s41537-023-00412-6

**Published:** 2023-11-20

**Authors:** Yuki Kikuchi, Yuji Yada, Yuji Otsuka, Fumiaki Ito, Hiroaki Tanifuji, Hiroshi Komatsu, Hiroaki Tomita

**Affiliations:** 1 Department of Psychiatry, Graduate School of Medicine, Tohoku University, Sendai, Miyagi Japan; 2 Department of Psychiatry, Kodama Hospital, Ishinomaki, Miyagi Japan; 3 Department of Psychiatry, Okayama Psychiatric Medical Center, Okayama, Japan; 4 Department of Psychiatry, Asahi General Hospital, Asahi, Japan; 5 National Hospital Organization Hanamaki Hospital, Hanamaki, Japan; 6 Department of Pharmacy, Kodama Hospital, Ishinomaki, Miyagi Japan; 7 Department of Psychiatry, Tohoku University Hospital, Sendai, Miyagi Japan

**Keywords:** Schizophrenia, Pharmacology

## Abstract

Clozapine-induced fever marks the beginning of its inflammatory and potentially life-threatening adverse effects, such as myocarditis. We retrospectively analyzed the correlation between clozapine titration rate and fever onset date in 254 Japanese patients, including 55 with treatment-resistant schizophrenia who developed clozapine-induced fever. Pearson’s product-moment correlation indicated a significant delay in the fever onset date with slower titration. Most fever onset cases occurred within 4 weeks, even with slow titration. Therefore, clinicians should remain vigilant in monitoring clozapine-induced fever within 4 weeks of clozapine initiation, regardless of the titration rate.

In Japan, clozapine-induced fever is reported at a high frequency of approximately 30%, following the protocol outlined in the Japanese package insert^1–3^. Even in the initial studies that were conducted in German-speaking countries, when clozapine titration experience was limited, it was found that approximately 5% of patients developed fever during the titration^4^. East Asians, including the Japanese, have a lower clozapine metabolizing capacity than Caucasians^5^. Therefore, to prevent clozapine-induced inflammatory adverse events, an international guideline recommends slow titration of clozapine for Asians^5^. The titration speed of clozapine by the protocol in the Japanese package insert is faster than that of this guideline, indicating that more inflammatory adverse effects may occur in Japanese patients^1^. Managing this fever is essential as it marks the beginning of clozapine’s inflammatory adverse effects, which in some cases leads to more serious conditions such as myocarditis and pneumonia^6^, and drug reaction with eosinophilia and systemic symptoms (DRESS) syndrome.

We recently published a study highlighting that slower clozapine titration in Japanese patients led to fewer inflammatory adverse effects^1^. Since then, we had an impression that slower titration does not only decrease the frequency but can also delay the onset of inflammatory adverse effects. To analyze the clozapine-induced inflammatory effects, we retrospectively reviewed the medical records of patients to investigate the relationship between the rate of clozapine titration and the onset of clozapine-induced fever.

We included 241 patients in this study, who were included in our previous analysis^1^, along with additional 13 patients newly introduced to clozapine at Kodama Hospital between February and September 2023, yielding a total of 254 patients. Medical records of patients with treatment-resistant schizophrenia who first started clozapine treatment at seven hospitals (Tohoku University Hospital, Miyagi Psychiatric Center, Hanamaki Hospital, Kunimidai Hospital, Aoba Hospital, Asahi General Hospital, and Kodama Hospital) were examined retrospectively. Among the 254 patients, 55 developed a fever of >38°C within 12 weeks of starting clozapine. The following information was collected from the medical records: age at clozapine initiation, sex, body mass index (BMI), smoking status, concomitant administration of valproic acid, concomitant administration of cytochrome P450 (CYP) 1A2 inhibitors (including fluvoxamine, amiodarone, ciprofloxacin, and oral contraceptives), clozapine initiation date, daily dose of clozapine, and fever onset date (FOD). The calculation of the clozapine titration rate (CTR) is described in our previous paper^1^. Briefly, we used the protocol recommended by the manufacturer in the Japanese package insert (increasing the dose by 25 mg/day every few days up to 200 mg/day in 3 weeks) as the reference (CTR = 1), and the CTR was determined relative to the reference by the following formula: CTR = cumulative clozapine dose up to the date the patient developed fever / cumulative clozapine dose up to the date the patient developed fever according to the reference protocol. Recently de Leon et al^5^. have proposed a guideline with slower titration rates to prevent inflammatory adverse events in patients of Asian origin with a CTR of 0.75, in comparison to current Japanese protocols. Accordingly, patients with a CTR >0.75 were classified as the faster-titration group (FG) and those with a CTR <0.75 were classified as the slower-titration group (SG). Past studies have shown that obesity and concomitant use of valproic acid significantly impact clozapine-induced inflammation^7,8^. These two factors inhibit the metabolism of clozapine, compromising the patient’s metabolism, leading to a poor metabolizer status. Therefore, we adjusted for these two confounding factors by stratifying patients into groups with or without overweight (BMI > 25) or valproate concomitant use and performed analyses in each group. All statistical analyses were performed using EZR (Saitama Medical Center, Jichi Medical University, Saitama, Japan)^9^. Differences in demographic data were analyzed with t-tests for continuous variables and Fisher’s exact tests for categorical variables. Pearson’s product-moment correlation coefficient was calculated for the correlation between CTR and FOD. The significance level was set at *P* < 0.05. Since the study design was a retrospective study using anonymous data, written informed consent was not obtained, but opt-out forms were presented on the bulletin boards or websites of the respective hospitals, and subjects who did not express the intent for exclusion were included in the study. The Tohoku University Hospital Ethics Review Board approved this study (Approval ID: 2022-1-1136).

There were no significant differences in factors known to impact baseline inflammation and clozapine metabolism: age, sex, BMI, concomitant valproic acid use, or smoking status between the FG and SG. There was no concomitant administration of CYP1A2 inhibitors (fluvoxamine, amiodarone, ciprofloxacin, or oral contraceptives) in any case. The mean CTR (standard deviation) was 0.96 (0.10) for the FG and 0.48 (0.16) for the SG (*P* < 0.001). The clozapine dose at onset of fever was 140.7 (36.4) mg/day for the FG and 67.5 (27.3) mg/day for the SG (*P* < 0.001). The mean FOD (standard deviation) was 15.7 (3.73) days in the FG and 18.2 (4.23) days in the SG (*P* < 0.05) (Table 1).

**Table 1:** Comparison of the faster-titration and slower-titration groups.

	Faster-titration group	Slower-titration group	Statistical test	*P*
Total, *n*	35	20		
Age, years, mean (SD)	40.1 (11.9)	39.8 (12.4)	*t* = 0.099, df = 53	0.92
Male, *n* (%)	18 (51)	13 (65)	Fisher’s exact test	0.40
Body mass index, kg/m^2^, mean (SD)	25.1 (5.47)	24.0 (5.31)	*t* = 0.756, df = 53	0.45
Overweight, *n* (%)	16 (46)	6 (30)	Fisher’s exact test	0.39
Clozapine titration rate, mean (SD)	0.96 (0.10)	0.48 (0.16)	*t* = 13.62, df = 53	<0.001
Clozapine dose at onset of fever, mg/day, mean (SD)	140.7 (36.4)	67.5 (27.3)	*t* = 7.809, df = 53	<0.001
Concomitant use of valproate, *n* (%)	10 (29)	7 (35)	Fisher’s exact test	0.76
Smoking, *n* (%)	3 (8.6)	1 (0.05)	Fisher’s exact test	1.00
Fever onset date, mean (SD)	15.7 (3.73)	18.2 (4.23)	*t* = −2.222, df = 53	0.031

*SD* standard deviation.

Pearson’s product-moment correlation indicated a significant correlation (correlation coefficient = −0.326, 95% confidence interval: −0.544 to −0.0666, *P* < 0.05), as shown in Fig. 1a. We then adjusted for overweight and concomitant valproate as risk factors of clozapine-induced inflammation. Patients were stratified by the presence or absence of these factors. In the SG patients without risk factors, the mean FOD was delayed to 20.1 days. SG patients with risk factors had a mean FOD of 16.8 days, similar to that of the FG patients (Supplementary Table 1). Pearson’s product-moment correlation indicated a stronger correlation in patients without risk factors (correlation coefficient = −0.65, 95% confidence interval: −0.845 to −0.303, *P* < 0.01) (Fig. 1b), and the correlation disappeared in patients with risk factors (correlation coefficient = −0.0692, 95% confidence interval: −0.398 to 0.275, *P* = 0.70) (Fig. 1c).

**Fig. 1:**
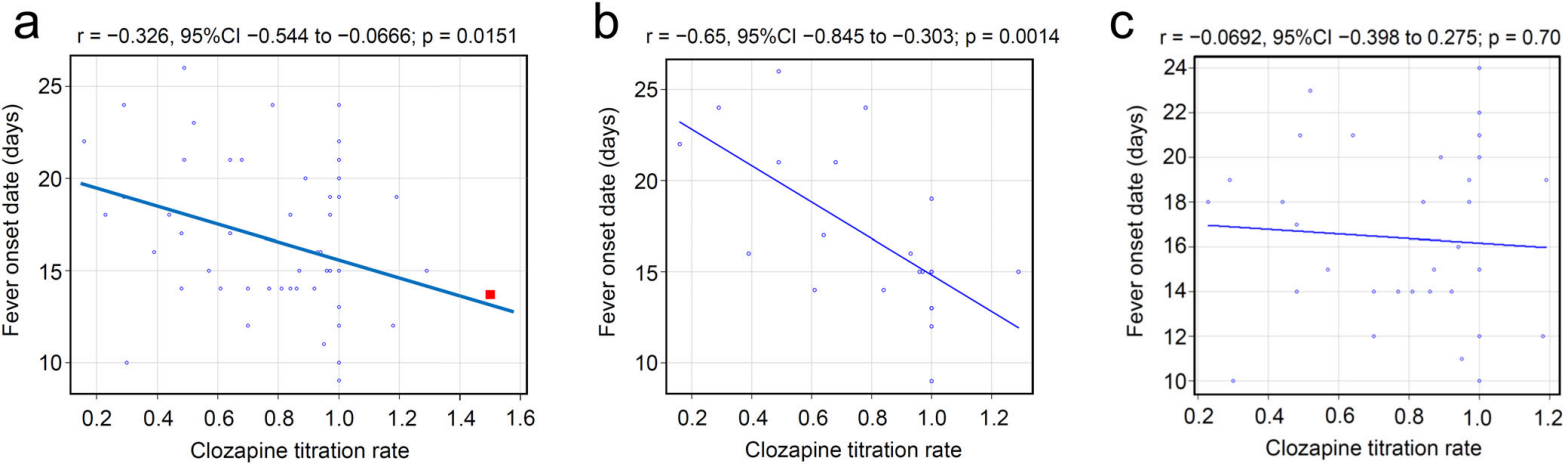
Correlation between fever onset date and clozapine titration rate. **a** A total of 55 cases. The data point from Pui-yin Chung et al. is shown as a red closed square; **b** 21 cases without risk factors; **c** 34 cases with risk factors. Risk factors refer to either BMI > 25 or concomitant use of valproic acid.

In this study, FOD was delayed by an average of approximately 2.5 days in the SG than in the FG. However, as shown in Fig. 1, the FOD varied from 9 to 24 days in the FG and from 10 to 26 days in the SG. Despite a trend indicating a delay in the FOD with slower titration, the correlation was weak, and it was difficult to linearly predict the FOD for individual cases. Thus, clinicians should remain vigilant about the onset of fever within 4 weeks of clozapine initiation, regardless of the titration rate. In contrast, it is interesting to note that the FOD is almost always within 4 weeks, even with a slow titration. Although there is a weak significant correlation between dose and the time to inflammatory response, the overall time course is similar across the first 4 weeks of treatment, suggesting the contribution of an immunological mechanism. The inflammatory response to clozapine is complex and a number of mechanisms have been proposed^10^.

The correlation between CTR and FOD was further demonstrated after adjusting for the confounding factors of overweight and concomitant use of valproate. The SG patients with risk factors tended to have fever as early as the FG patients. This suggests that even with slow titration, patients with risk factors may have elevated blood levels of clozapine, owing to their poor metabolizer status. In FG patients, the lack of change in FOD with risk factors suggests that the rapid dose escalation itself is a major risk, whereas the effects of overweight and concomitant valproic acid are negligible.

A limitation of this study was the relatively small number of cases, necessitating validation with a larger number of cases. Second, as this is a study based in Japan, our results need to be validated in other ethnic groups. Third, since we included a wide range of febrile cases, it is possible that some cases are not directly relevant to clozapine. Fourth, the CTR range in this study is limited to 1.0 or less. As CTR of 1 in this study was based on the protocol in the Japanese package insert, it is reasonable that clinicians rarely exceeded a CTR of 1, and may have considered the risk of adverse effects and chosen lower CTR in some cases. It would be interesting to see if there is a correlation between CTR and FOD in the range of CTR > 1. Although this research design may not be applicable in Japan, due to the risk of adverse effects, examining the correlation between CTR and FOD in the U.S. and Australia may be relevant, where dose escalation above CTR=1.5 is standard practice^5,11^. Finally, we would like to point out that weekly C-reactive protein (CRP) monitoring during clozapine initiation, as recommended by the international guideline^5^, may prevent fever development^12^. CRP monitoring was not done systematically. It is possible that these fevers could have been avoided by titration reduction as soon as the CRP elevation was observed during weekly CRP monitoring.

To the best of our knowledge, only one report described the rate of clozapine dose titration and date of onset of clozapine-induced fever based on a small sample-sized observation^13^. The study reported that 31 Asian patients with clozapine-induced fever had a mean titration rate of 99.8 mg/week (1.50 in terms of CTR) and a mean onset of fever of 13.7 days, which was concordant with our regression line (Fig. 1a). Thus, the current study established a correlation between the titration rate and the onset of inflammatory adverse effects through a systematic survey.

### Supplementary information

Supplementary Table 1

## Data Availability

The data are not publicly available because they contain information that could compromise the research participants’ privacy/consent.
