# Supplementary material for: Slower clozapine titration is associated with delayed onset of clozapine-induced fever among Japanese patients with schizophrenia
Source: Schizophrenia (Heidelb). 2023 Nov 20;9(1):82. doi: 10.1038/s41537-023-00412-6 (PMC10661360; doi:10.1038/s41537-023-00412-6)
Supplement: Supplementary file 1 — Supplementary Table 1 [file 41537_2023_412_MOESM1_ESM.docx]

| **Supplementary Table 1** |  |  |  |  |  |
| --- | --- | --- | --- | --- | --- |
| Differences in fever onset date by risk factors in each group | | |  |  |  |
|  |  | With risk factors | Without risk factors | Statistical test | P |
| Fever onset date, mean (SD) | All cases | 16.4 (3.83) | 17.0 (4.48) | t = 0.503, df = 53 | 0.62 |
|  | Slower-titration group | 16.8 (3.93) | 20.1 (4.12) | t = 1.80, df = 18 | 0.088 |
|  | Faster-titration group | 16.1 (3.85) | 15.0 (3.56) | t = −0.868, df = 33 | 0.39 |
| Abbreviation: SD, standard deviation. | |  |  |  |  |
